# Palinopsia associated with the CDK4/6 inhibitor ribociclib during the first-line treatment of metastatic breast cancer: two case reports

**DOI:** 10.3389/fonc.2024.1430341

**Published:** 2024-12-19

**Authors:** Tamara Martos, Marta Saint-Gerons, Laura Masfarre, Maria Castro-Henriques, Maria Martinez-Garcia, Sonia Servitja, Joan Albanell

**Affiliations:** Hospital del Mar, Parc de Salut Mar, Barcelona, Spain

**Keywords:** palinopsias, ribociclib, breast cancer, adverse events, ophthalmologic toxicity

## Abstract

The most frequently used standard treatment for hormone receptor (HR)-positive, human epidermal growth factor receptor 2 (HER2)-negative metastatic breast cancer patients consists of a CDK4/6 inhibitor (abemaciclib, ribociclib, or palbociclib) combined with endocrine therapy. Despite CDK4/6 inhibitors being part of routine care in the last few years, new adverse events continue to be reported. Here, we report two cases of palinopsia, a rare neurological visual disturbance that refers to the persistence or recurrence of a visual image after the removal of visual stimuli in patients treated with ribociclib and letrozole. Neuro-ophthalmological assessments and brain MRIs did not find any organic cause. However, palinopsia was related in a time- and dose-dependent manner to the intake of ribociclib. Following a one-level dose reduction of ribociclib, palinopsia was mild and well tolerated. Both patients continued the treatment with ribociclib, with one of them for almost 2 years. Based on the identification of two cases in our hospital in a short period of time, it is tempting to suggest that ribociclib-related palinopsias may not be uncommon. We propose that physicians should be aware of this ribociclib-associated adverse event. Patients presenting this symptom should undergo a routine workup (neuro-ophthalmological assessment and brain MRI) and, if negative, be reassured of its relation with ribociclib as well as the safety of continuing on this drug.

## Highlights

- For the first time, palinopsias had been described to be related to ribociclib as a rare adverse event in patients with metastatic breast cancer.

## Introduction

Breast cancer accounts for approximately 30% of female cancers and has a mortality-to-incidence ratio of 15%. Approximately 20% of the patients suffer a metastatic relapse after the treatment for early breast cancer, and approximately 5% have *de novo* stage IV metastatic disease. Hormone receptor (HR)-positive, human epidermal growth factor receptor 2 (HER2)-negative disease is the most common subtype of breast cancer. Abemaciclib, palbociclib, and ribociclib are the three CDK4/6 inhibitors approved for HR+, HER2-negative advanced breast cancer in combination with endocrine therapy (1–3). The combination of endocrine therapy with ribociclib, abemaciclib, and ribociclib has become the first-line standard of care (SOC) for locally advanced inoperable or metastatic HR+, HER2− breast cancer patients due to its benefits in progression-free survival and overall survival. The overall adverse event (AE) profile of the different CDK4/6 inhibitors is well known. However, each CDK4/6 inhibitor (CDK4/6i) has distinct AEs, mainly due to their different degrees of specificity and potency in terms of inhibition of CDK4 and CDK6. With the widespread use of these agents, rare toxicities of CDK4/6 inhibitors are being reported. To our knowledge, neurological AEs, including palinopsia, due to CDK4/6 inhibitors have not been reported to date. Here, we report two cases of women who described palinopsias during ribociclib therapy in a time- and dose-dependent manner.

## Case 1

In 2002, a 46-year-old woman with a history of Raynaud’s syndrome and hypothyroidism was diagnosed with HR-positive [estrogen receptor (ER) 95%, progesterone receptor (PR) 95%] HER2-negative low-grade invasive cancer (NOS) of the right breast cancer (pT1cpN1aM0). The primary treatment was a right radical mastectomy. The patient received adjuvant chemotherapy (docetaxel, cyclophosphamide, and doxorubicin for six cycles), irradiation of the affected breast and supra- and infraclavicular nodes, and adjuvant endocrine therapy with tamoxifen for 7 years.

The patient remained free of relapse until August 2021. At this point, the patient consulted a general practitioner for hip pain. A bone scintigraphy and a CT scan were performed, which identified a left iliac lesion suspicious of bone metastases and unspecific lung nodes. A bone biopsy revealed invasive breast cancer (NOS, ER+ 100%, PR+ 100%, HER2-negative). In September 2021, she was started on ribociclib 600 mg/day (three 200-mg tablets daily for 3 weeks every 4 weeks) plus letrozole (2.5 mg/day continuous).

**Figure 1:**
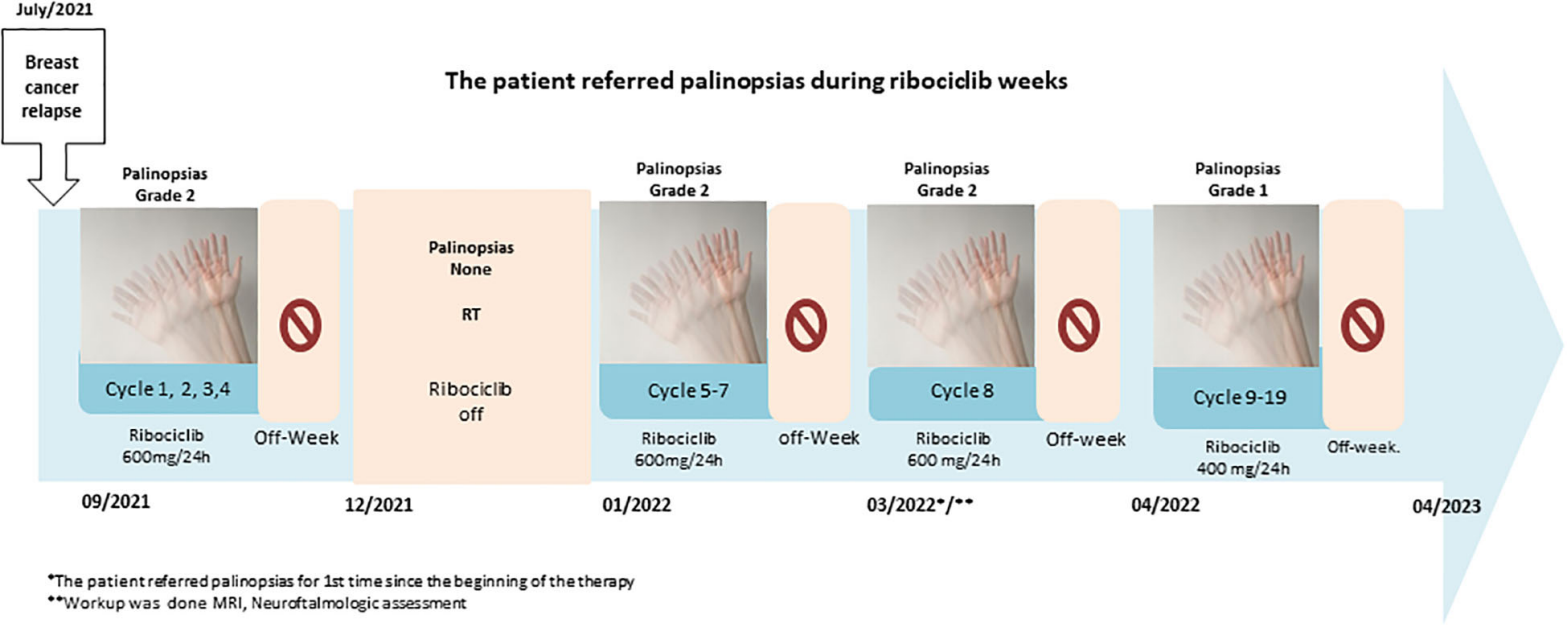
Cronologic symptoms in case 1 during the intake of Riboclib. *The patient referred palinopsias for 1st time since the beginning of the therapy. **Workup was done MRI, Neuroftalmologic assessment.

In November 2021, the patient came to our hospital. We performed a PET/TC scan that did not reveal additional metabolic foci of disease, other than the known left iliac lesions. We continued ribociclib and letrozole and planned radiation therapy [stereotactic body radiation therapy (SBRT)] to the bone lesion (January 2022). Ribociclib was discontinued for three consecutive weeks to avoid an overlap with radiation therapy. In March 2022, the patient referred persistence of small motion visual images in dim conditions after the removal of visual stimuli, i.e., palinopsia. Retrospectively, the patient acknowledged that palinopsia started in cycle 1 of ribociclib, from day 1 to day 21, and disappeared in the week off ribociclib (**Figure 1**) It was moderately disturbing, and the pattern was the same for each cycle. As palinopsia was absent in the 3-week period while off ribociclib (but not letrozole) when she had the radiation therapy, the patient suspected a drug-related cause and discussed it with us. A workup was performed, including a neurological examination and a neuro-ophthalmological assessment. Her best-corrected visual acuity was 20/20 in both eyes (OU). She had equal and reactive pupils with no relative afferent pupillary defect. Slit-lamp biomicroscopy, intraocular pressure, and funduscopy revealed normal OU. Optical coherence tomography showed a normal peripapillary retinal nerve fiber layer, macular, and ganglion cell layer analysis of OU. Automated visual fields revealed a normal field of OU, as did a laboratory blood test and a brain MRI. None of them revealed any organic cause of palinopsia. The chronology of symptoms and ribociclib intake and the absence of alterations in complementary tests guide the etiology of palinopsia as a rare adverse event related to ribociclib. In April 2022, after careful discussion with the patient, the ribociclib dose was lowered to 400 mg/day. Following dose reduction, palinopsia disappeared until July 2022. The palinopsia reappeared at a very light degree and low frequency. Since then, the patient has been on ribociclib therapy at 400 mg and has very mild, non-daily palinopsias and no evidence of disease progression (October 2024).

## Case 2

In December 2022, a 28-year-old premenopausal woman with a history of lymphoma, treated with polychemotherapy (scheme REPOCH), radiotherapy, and surgery of residual mass in 2018, was diagnosed with breast cancer. She had a metastatic invasive high grade with HR-positive (ER 99%, PR 0%) and HER2-negative [immunohistochemistry (IHC) 0+] breast cancer. She had cT3cN1M1 disease. Distant metastasis was located in the brain (parietal lobe and both left and right cerebellar hemispheres) and peritoneum. A biopsy of the peritoneal node confirmed metastases from breast cancer. Brain metastasis was not amenable to surgical resection and did not require immediate radiation therapy In January 2023, the patient started first-line systemic therapy with luteinizing hormone-releasing hormone (LHRH) analogs (goserelin 3.6 mg sc q28days), letrozole (2.5 mg q24h), and ribociclib (three 200-mg tablets daily for 3 weeks followed by 1 week off, every 4 weeks). The patient reported that on the third day of ribociclib the appearance of mild abnormal visual symptoms related to motion when the visual field was no longer there (palinopsia). These images were present every day during bright light conditions or at night. Palinopsia disappeared the first day of the week off ribociclib, and no neurological additional disturbances were reported by the patient. A neuro-ophthalmologist conducted a visual exam: the patient’s best-corrected visual acuity was 20/20 in both eyes. She had equal and reactive pupils with no relative afferent pupillary defect. Slit-lamp biomicroscopy, intraocular pressure, and funduscopy were normal OU. Optical coherence tomography showed a normal peripapillary retinal nerve fiber layer, macular, and ganglion cell layer analysis of OU. Automated visual fields revealed a normal field of OU. Brain metastases were not considered as a cause of the patient’s palinopsia. According to the clear temporal relationship of palinopsia with ribociclib intake and our experience with case 1, we considered this an AE of ribociclib. The patient continued the treatment with ribociclib and endocrine therapy. Palinopsia persisted at the same mild intensity during the on-ribociclib weeks, without any impact on her routine activities.

## Discussion

Since the approval of CDK4/6i for HR+, HER2-negative metastatic breast cancer (mBC), these drugs have become the first line of therapy in combination with aromatase inhibitor or fulvestrant. Usually, CDK4/6is are well tolerated, and AEs are generally manageable with dose modification and supportive care measures. The most common AE reported of palbociclib and ribociclib is neutropenia, while the most common AE reported of abemaciclib is diarrhea. No neurological visual adverse events had been described related to CDK4/6i (2, 4).

Palinopsia is a rare neurological visual disturbance that refers to the persistence or recurrence of a visual image after the removal of visual stimuli. Although the exact physiopathology of this phenomenon remains unknown, pathophysiological causes include dysfunction of the coordinate systems of the parietal lobes (5) and the pathways involving processing and feedback of light and motion with alteration of serotonergic activity (6). It is classified into two subgroups: hallucinatory and illusionary. Hallucinatory palinopsia indicates a central nervous system (CNS) alteration, and the causes include neoplasms, seizures, arteriovenous malformation, stroke, or infection. However, illusory palinopsia is strikingly dependent on external light or the motion of an object and is characterized by prolonged indistinct or unformed perseverated images or light (longer than physiological afterimages). The causes include drugs, migraines, menstrual cycle, hallucinogen persisting perception disorder, head injuries, or metabolic diseases (7–11).

Case reports had been published of palinopsias related to prescription drugs: trazodone, nefazodone, mirtazapine, topiramate, clomiphene, oral contraceptives, and risperidone (12–14). Palinopsia usually occurs after introducing the aforementioned drugs or increasing their dose, and it resolves after discontinuation. The pathophysiology of drug-induced palinopsia is based on alterations in neurotransmitters and their receptors, such as the serotonergic system or disruption in GABAergic transmission, which is facilitated by the 5HT2a receptor (15, 16). Estrogens interact with neurotransmitters, including the brain’s cholinergic and serotonergic systems, and hormonal therapies could interfere with the normal CNS process involved concerning the visual field. However, CDK4/6is are not cell-specific, so these agents can interfere not only with the cell cycle of cancer cells but also with the cell cycle of healthy cells in brain tissue. Nevertheless, further investigation is still required to understand the exact mechanisms as well as potential risk factors.

Patients with palinopsias may present to general practitioners, neurologists, or ophthalmologists with visual symptoms, which may be misdiagnosed. Palinopsias need proper ophthalmological and neurological physical exams. Visual acuity, pupil, tonometry, extraocular movement, and external exams are needed, although physical exam and workup are almost always non-contributory, and diagnosis is largely based on information from the clinical history (14).

The two patients described above experienced visual abnormalities early during the ribociclib therapy. The differential diagnoses included CNS spread or toxicity related to aromatase inhibitors. A neurological exhaustive physical exam by a specialized oncologist disregarded other neurological symptoms, and an MRI was performed without evidence of metastases in CNS in the first case and absence of deterioration in the second case. A neuro-ophthalmologist performed a visual exam and did not find ocular pathology. The close temporal relationship between the therapeutic regimen with ribociclib and the reversibility after the ribociclib withdrawal in our patients suggests the direct involvement of palinopsias and CDK4/6i. The woman described in the second case has CNS metastases, but she had not experienced such episodes until the beginning of CDK4/6i therapy. Her abnormal visual alterations started while on ribociclib therapy and disappeared when the therapy was discontinued. In this case, the patient had brain M1; however, the chronology and the clinical course of these symptoms guided our clinical suspicion that palinopsias could be related to ribociclib.

Palinopsia should be distinguished from physiological afterimages or toxicity related to aromatase inhibitors (AIs). Physiological afterimages are a common and benign phenomenon that appears after viewing a bright stimulus and shifting visual focus is thought to derive mainly from photobleaching of the retina, although newer evidence indicates a contribution from cerebral processes (17–19).

The incidence of ocular toxicity related to AI is quite variable. It had been described previously as dry eyes, Sjögren’s syndrome, and ocular surface abnormalities in AI-treated breast cancer patients. Several clinical studies evidenced that hormonal therapy with AI impacts the ocular surface and Meibomian glands. Retinopathy and maculopathy were reported, which could present as blurred vision or ocular pain. These symptoms are accompanied by alterations in ocular coherence tomography in the foveal and parafoveal areas (20).

Ribociclib combined with AI is the first-line therapy in HR-positive, HER2-negative advanced breast cancer since the data published in MONALEESA-2 and MONALEESA-7 trials show substantial benefit in progression-free survival (PFS) and overall survival (OS) compared with hormonal therapy alone. In the pivotal trials, no visual alterations were reported related to CDK4/6i therapy. However, since the approval by Health Authorities worldwide, ribociclib has been used widely, which allows clinicians to identify new, rare adverse events like cutaneous toxicity (21, 22); but to our knowledge, no case of ribociclib-related palinopsia has been described.

## Data Availability

The original contributions presented in the study are included in the article/supplementary material. Further inquiries can be directed to the corresponding author.
